# Reactive Blending of Modified Thermoplastic Starch Chlorhexidine Gluconate and Poly(butylene succinate) Blending with Epoxy Compatibilizer

**DOI:** 10.3390/polym15163487

**Published:** 2023-08-21

**Authors:** Nanthicha Thajai, Pornchai Rachtanapun, Sarinthip Thanakkasaranee, Winita Punyodom, Patnarin Worajittiphon, Yuthana Phimolsiripol, Noppol Leksawasdi, Sukunya Ross, Pensak Jantrawut, Kittisak Jantanasakulwong

**Affiliations:** 1 Nanoscience and Nanotechnology (International Program/Interdisciplinary), Faculty of Science, Chiang Mai University, Chiang Mai 50200, Thailand; nanthicha581@gmail.com; 2 School of Agro-Industry, Faculty of Agro-Industry, Chiang Mai University, Mae-Hea, Mueang, Chiang Mai 50100, Thailand; pornchai.r@cmu.ac.th (P.R.); sarinthip.t@cmu.ac.th (S.T.); yuthana.p@cmu.ac.th (Y.P.); noppol.l@cmu.ac.th (N.L.); 3 Center of Excellent in Agro Bio-Circular-Green Industry (Agro BCG), Faculty of Agro-Industry, Chiang Mai University, Chiang Mai 50100, Thailand; 4 Center of Excellence in Materials Science and Technology, Faculty of Science, Chiang Mai University, Chiang Mai 50200, Thailand; winita.punyodom@cmu.ac.th (W.P.); patnarin.w@cmu.ac.th (P.W.); 5 Department of Chemistry, Faculty of Science, Chiang Mai University, Chiang Mai 50200, Thailand; 6 Department of Chemistry, Faculty of Science, Naresuan University, Phitsanulok 65000, Thailand; sukunyaj@nu.ac.th; 7 Department of Pharmaceutical Sciences, Faculty of Pharmacy, Chiang Mai University, Muang, Chiang Mai 50200, Thailand; pensak.amuamu@gmail.com

**Keywords:** biopolymer, modified starch, chlorhexidine gluconate, poly (butylene succinate), epoxy resin

## Abstract

Biodegradable starch-based polymers were developed by melt-blending modified thermoplastic starch (MTPS) with poly(butylene succinate) (PBS) blended with epoxy resin (Er). A modified thermoplastic starch blend with chlorhexidine gluconate (MTPSCh) was prepared by melt-blending cassava starch with glycerol and chlorhexidine gluconate (CHG) 1.0% wt. The Er was melt-blended with PBS (PBSE) at concentrations of 0.50%, 1.0%, 2.5%, and 5.0% (wt%/wt%). The mechanical properties, water resistance, and morphology of the MTPSCh/PBSE blends were investigated. The MTPSCh/PBSE2.5% blend showed an improvement in tensile strength (8.1 MPa) and elongation at break (86%) compared to the TPSCh/PBS blend (2.6 MPa and 53%, respectively). In addition, water contact angle measurements indicated an increase in the hydrophobicity of the MTPSCh/PBSE blends. Thermogravimetric analysis showed an improvement in thermal stability when PBS was added to the MTPSCh blends. Fourier transform infrared spectroscopy data confirmed a new reaction between the amino groups of CHG in MTPSCh and the epoxy groups of Er in PBSE, which improved the interfacial adhesion of the MTPSCh/PBSE blends. This reaction improved the mechanical properties, water resistance, morphology, and thermal stability of the TPSCh/PBSE blends.

## 1. Introduction

Researchers have attempted to develop alternative materials for natural and biodegradable polymers from renewable resources to reduce the use of petroleum-derived plastics [1]. Biopolymers are major materials in the development of biodegradable film for packaging and agricultural and medical applications; they include polysaccharides, polypeptides, and polynucleotides. Polysaccharides are formed by the glycosidic linkage of monosaccharides. Starch, cellulose, chitosan, pectin, glycogen, and alginate are polysaccharide base materials. Biopolymers with an amide linkage are effective biomaterials for reactions with other reactive functional groups, such as keratin, sericin, fibroin, collagen, gelatin, and polypeptides [2]. Synthesis is a technique used to develop biopolymers via polymerization and the chemical modification of natural monomer materials that present inherent biodegradability, biocompatibility, and excellent mechanical properties. Among biopolymers, starch is one of the most promising candidates for the preparation of packaging applications. Starch has been used as a base film and filler due to its biodegradable ability, low cost, and renewability. Starch forms strong intermolecular hydrogen bonds and degrades before melting. It can be converted into thermoplastic starch (TPS) by gelatinization and plasticization. The preparation of TPS requires the incorporation of plasticizers such as sorbitol, glucose, and glycerol with high temperature and shear force. Glycerol (25–40%) is an effective plasticizer to develop TPS [3]. In particular, TPS has been extensively used for the development of bioplastic films and packaging because of its cost-effectiveness, complete biodegradability, and natural abundance [4]. TPS shows desirable properties, including the fact that it is colorless, odorless, and non-toxic [5]. However, TPS has several significant disadvantages, such as poor mechanical properties, low thermal processing ability, high water sensitivity, and plasticizer migration [6,7]. Several studies have investigated modified starch to improve the water sensitivity and reactivity of starch [8,9]. Modifications of starch are commonly conducted to alter its hydrophilicity by replacing the hydroxyl groups with chemically active groups. Cationic, anionic, and nonionic materials have been widely used for starch modification as they can be attached to the hydroxyl groups of starch. Modified cationic starches are widely used in the global paper industry, but few studies have investigated their application in preparing TPS [10,11,12,13]. It has been determined that the processability, water resistance, and mechanical properties of TPS can be improved by blending it with other completely biodegradable materials. To overcome the limitations of TPS, the blending of TPS with bioplastics, such as polylactic acid (PLA), poly (butylene succinate) (PBS), and poly(butylene adipate co-terephthalate) (PBAT), has been studied [14,15]. Reactive blending is an effective technique to improve biodegradable material properties [16]. TPS is usually blended with other natural materials, polymers, and nanoparticles, etc. [17]. Agricultural waste has been blended with TPS extensively due to its low cost, high added value, wide range of sources, and abundant materials. However, the incompatibility of the TPS blend provides poor morphology, interfacial tension, water resistance, and mechanical properties due to its structure and hydrophilic properties. It is important to develop TPS properties using starch modification, synthesis, composites, and reactive blending techniques. The development of new biopolymers should be targeted towards emphasizing degradation ability and environmental assessments.

In recent years, bioplastics research has been focused on property improvements, such as PLA, PBS, and PBAT. Increasing the amount of bioplastics development that utilizes renewable resources has become a top priority. However, high costs and the inadequacy of the mechanical properties prevent the use of bioplastics in a wide range of applications. Among these bioplastics, PLA is a biobased fully biodegradable polyester that is derived from agricultural materials. PLA shows high transparency, stiffness, tensile strength, biocompatibility, and processability. PLA has been limited because of its low degradation rate and high brittleness. PBAT is a ductile thermoplastic polyester with high mechanical properties and flexibility and is similar to polyethylene. PBAT has usually been used to toughen biopolymers with the addition of a reactive compatibilizer. Several studies have investigated a TPS blend with PBAT [18,19]. PBS is an aliphatic polyester that is polymerized from butanediol and succinic acid and can be derived from petroleum-based and bio-based renewable resources [20,21]. The mechanical properties and processability of PBS are similar to those of polyethylene. PBS is a commercial bioplastic that can be used to prepare plastic bags, films, and injection-molded packaging. The low temperature (130–160 °C) used to process PBS is lower than that for PLA. PBS has excellent properties, including good tensile strength (18–30 MPa), elongation (20–500%), ductility, biodegradability, and processing capabilities [22,23], compared with low-density polyethylene (tensile strength 10 MPa and elongation 500%) [24]. The use of bioplastics in the preparation of biopolymer blends is an effective method with which to develop the mechanical and water resistance properties of the blend. The addition of a compatibilizer is the key to improving the compatibility between the biopolymer and bioplastic blend.

Epoxy resin (Er) is a thermoset polymer. Its network structure is formed via a reaction between the epoxy groups of an epoxy monomer and the amino groups of a hardener. Er shows excellent mechanical properties, chemical resistance, low cost, and easy processing. Er is used as a matrix material for composites that are used in automotive and construction materials and in the electronics, coating, and aerospace fields [25]. The toughening of Er without reinforced materials is a major topic of research. Nanometal particle-reinforced Er has been extensively investigated [26,27]. Bisphenol A diglycidyl is an epoxy monomer part of Er. It is a reactive crosslinking agent that is usually used in polymers, such as polycarbonate [28], polycaprolactone [29], polyamine [30], and polylactic acid [31]. Er consists of reactive epoxide groups that crosslink with other functional groups, such as hydroxyl and carboxyl groups (–COOH), leading to improved morphology as well as the enhanced melting and crystallization properties of polymer blends [32,33]. However, the blending of Er and PBS has not been widely researched. In addition, the improvement of the mechanical, water resistance, and antimicrobial properties of starch blends modified with chlorhexidine gluconate (CHG), PBS, and Er has not been reported. 

Therefore, the aim of this study was to develop MTPS from cassava starch blends with CHG, PBS, and Er to improve the mechanical properties, water resistance, morphology, thermal stability, and reactivity of the blends for medical, agricultural, and packaging applications. MTPS was used as the main matrix polymer because of its hydrophobicity and polarity, whereas CHG was mixed with MTPS to provide amino groups during the melt blending of the modified thermoplastic starch. Er was used to improve crosslinking and provide epoxy groups to PBSE. The reaction between the amino groups of MTPSCh and the epoxy groups of PBSE may induce interfacial crosslinking and improve the properties of the MTPSCh/PBSE blend. 

## 2. Materials and Methods

### 2.1. Materials

Modified starch (modified tapioca starch, EXCELCAT^®^30) with a moisture content of 13 (wt%/wt%), DS of 0.028–0.032, and pH of 4.5–6.0 was purchased from Siam Modified Starch Co., Ltd., Pathum Thani, Thailand. Glycerol (99%) was purchased from Union Science Co. Ltd., Chiang Mai, Thailand. PBS pellets (BioPBS™ FD92PM, density 1.24 g/cm^3^, MFI 4 g/10 min at 190 °C) were purchased from PTT Global Chemical Pub Co., Ltd., Bangkok, Thailand. CHG (20% solution) was purchased from S. Tong Chemical Co., Ltd., Chiang Mai, Thailand. Er (diglycidyl ether of bisphenol, grade A 0302) was purchased from Easy Resin Co., Ltd., Nonthaburi, Thailand.

### 2.2. Sample Preparation

MTPS was prepared by mixing the modified starch with glycerol (70/30 wt%/wt%) and 500 mL of distilled water in a water bath (WNB 14 L0, Memmert GmbH + Co. KG, Bavaria, Germany) with an overhead stirrer at 70 °C for 30 min. CHG at 1.0 wt%/wt% was added to the MTPS as an additive to prepare MTPSCh. Distilled water in the premixed starch was evaporated in a hot air oven at 50 °C for 24 h. PBSE was prepared by melt-blending PBS with Er at 0.5%, 1.0%, 2.5%, and 5.0% (wt%/wt%) using a two-roll mill (PI-140, Pirom-Olarn Co. Ltd., Bangkok, Thailand) at 130 °C for 15 min. The melt blending of MTPSCh (90%) and PBSE (10%) were performed using a two-roll mill at 130 °C for 5 min. The samples were compressed into sheets using a hot compress at 130 °C for 5 min to evaluate their properties. The concentrations and codenames of the samples are listed in Table 1. 

### 2.3. Tensile Properties

The tensile properties of the samples were measured following JISK-6251-7 using a tensile tester (Model H1KS, Hounsfield test equipment, Surrey, England) with a gap length of 10 mm and crosshead speed of 10 mm/min. The specimens were prepared as sheets with dimensions of a 5 mm width, 30 mm length, and 1 mm thickness. Five specimens from each of the samples were conditioned at 25 °C and 50 ± 2% RH for 24 h. The stress–strain curve, maximum force, and elongation at break were recorded. 

### 2.4. Contact Angle

Water contact angles were measured using a drop shape analyzer (DSA30E, Krüss Co., Ltd., Hamburg, Germany). A droplet of water was dropped onto the surface of the sample, and images were recorded automatically every minute for 10 min. The samples were prepared as sheets (1 × 1 cm^2^) via hot-compression molding at 130 °C for 5 min. The surface of the hot-compressed sample was used to observe the water droplet contact angle. All the samples were conditioned at a relative humidity of 50 ± 2% RH for 24 h.

### 2.5. Scanning Electron Microscopy (SEM)

The morphology and fractured surfaces of the samples were analyzed using SEM (JSM-IT300LV model, JEOL Co., Ltd., Tokyo, Japan). The samples were broken in liquid nitrogen, evaporated in a hot air oven (60 °C) for 1 h, and then coated with a thin layer of gold. The fractured samples were observed at 15 kV under a vacuum. 

### 2.6. Thermogravimetric Analysis (TGA)

TGA of all the samples was performed using a thermogravimetric analyzer (Mettler-Toledo STARe system TGA/DSC3+, Greifensee, Switzerland) at 10 °C/min under a nitrogen atmosphere from 25 to 550 °C. A sample weight of 10–15 mg was used to evaluate the degradation characteristics of the samples. 

### 2.7. Reaction Mechanism

Fourier transform infrared (FTIR) spectra were measured to study the reaction mechanism of the samples using an FTIR spectrometer (FT/IR-4700, Jasco Corp., Tokyo, Japan) with a resolution of 4 cm^−1^ in the range of 600–4000 cm^−1^. The samples were prepared as films via hot-compression molding at 130 °C for 5 min.

### 2.8. Statistical Analysis

One-way ANOVA with SPSS software (SPSS version 17, Armonk, NY, USA) was used to analyze the results. The differences (*p* < 0.05) were estimated using the Duncan test.

## 3. Results and Discussion

### 3.1. Reaction Mechanism

**Figure 1:**
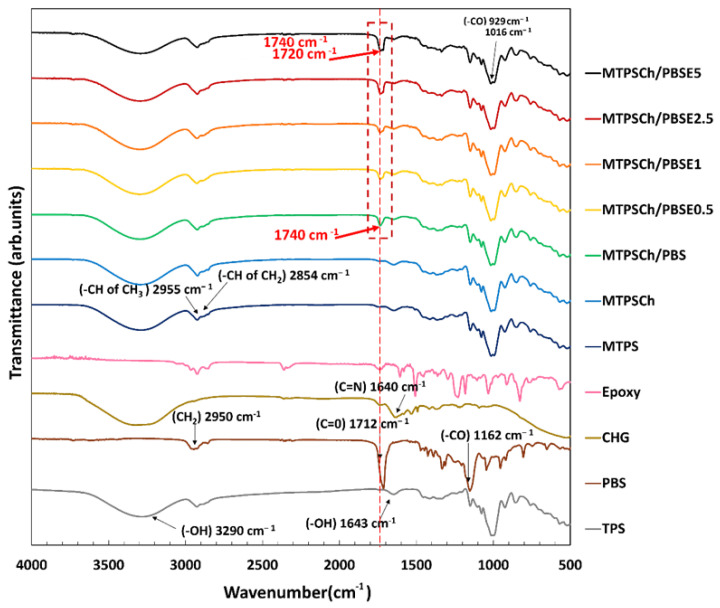
FTIR spectra of TPS, PBS, CHG, MTPS, MTPSCh, MTPSCh/PBS, and MTPSCh/PBSE blends with Er at 0.5–5.0%.

FTIR is an important instrument in the study of the structures and functional group reactions in polymer blends. It was used to characterize the structures and reaction mechanisms of the MTPS and PBS blends with CHG and the Er compatibilizer addition. The FTIR spectra of the TPS, PBS, CHG, MTPS, MTPSCh, MTPSCh/PBS, and MTPSCh/PBSE blends with 0.5–5.0% Er are shown in Figure 1. TPS and MTPS exhibited main peak bands at 3290, 1643, 1016, and 929 cm^−1^, which corresponded with the hydroxyl group (–OH), vibrations of the bending hydroxyl group (–OH) and the scissoring of two –CO stretching bands, respectively [34,35,36,37]. CHG presented characteristic peaks at 1580 (N–H), 1640 (C=N), 1095, and 1155 cm^−1^ (C–O–C of the chloride-bonded aromatic ring) [38,39,40]. In the FTIR spectrum of PBS, the characteristic peaks were observed at 2950, 1712, and 1162 cm^−1^, which correspond with the alkyl (–CH_2_), carbonyl (C=O), and (–CO) stretching vibrations of PBS, respectively [41,42]. The MTPSCh blend with PBS and PBSE exhibited the combination spectra of MTPS, CHG, PBS, and PBSE. The intensity of the –CO stretching main peak of MTPS at 1016 cm^−1^ was used to normalize the spectra of the blends. The MTPCh/PBS showed a shifting peak of C=O PBS at 1712 cm^–1^ to 1740 cm^–1^, which indicated the stretching of a new C=O vibration from the strong reaction between the carboxyl end groups (–COOH) of PBS and the amino groups (–NH) of CHG in MTPSCh. This reaction improved the elongation properties of MTPCh/PBS. MTPCh/PBSE showed a new peak at 1720 cm^–1^ and an overlap peak with increased intensity at 1720–1740 cm^–1^, which was attributed to the stretching of an ester carbonyl owing to the reactions of the epoxy groups in the PBSE and –COOH end groups of PBS with the amino groups of CHG in MTPSCh. This reaction improved the mechanical properties, water resistance, morphology, and thermal properties of the blends. The improvement in the properties of polymer blends owing to the reaction between the epoxy groups and amino groups was reported previously [36,43,44]. 

### 3.2. Mechanical Properties

**Figure 2:**
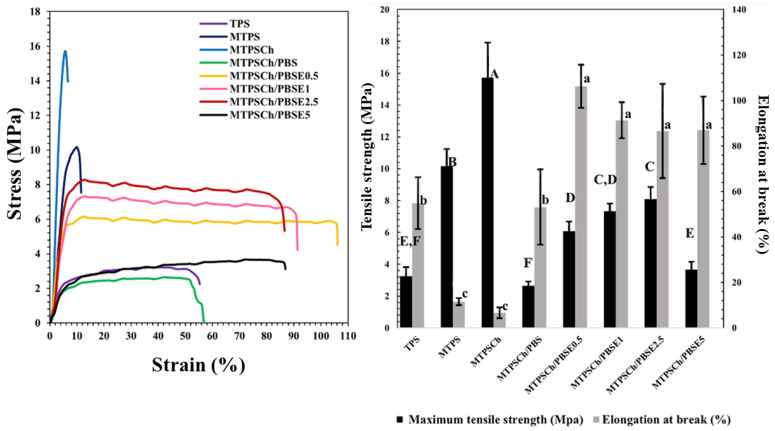
Tensile properties of TPS, MTPS, MTPSCh, MTPSCh/PBS, and MTPSCh/PBSE blends with 0.5–5.0% Er. Mean values of the elongation at break (lowercase letters) and maximum tensile strength (uppercase letters) differ significantly (*p* < 0.05).

The tensile properties of TPS, MTPS, MTPSCh, and MTPSCh melt-blended with PBS and PBSE at 0.5%, 1.0%, 2.5%, and 5.0% of Er are presented in Figure 2. The tensile modulus was calculated from the slope of the stress–strain curve. The tensile moduli the of TPS, MTPS, and MTPSCh samples were 12, 105, and 337 MPa, while the MTPSCh/PBSE blends with the Er 0, 0.5, 1, 2.5, and 5% samples were 26, 118, 85, 109, and 27 MPa, respectively. MTPSCh showed the highest tensile modulus due to the high crosslinking between MTPS and CHG. The tensile strength of MTPS (10.2 MPa) was higher than that of TPS (3.3 MPa). However, the elongation at break of MTPS (1.5%) was lower than that of TPS (54.8%). Upon adding CHG 1.0% to MTPS, the tensile strength increased to 15.7 MPa, and the elongation at break decreased to 6.7% owing to the formation of a crosslinking network of starch with CHG. The reaction-induced crosslinking of TPS after the CHG addition was reported previously [38]. The MTPSCh/PBS blend had a reduced tensile strength of 2.6 MPa and an increased elongation at break of 53.1%, owing to the flexibility of the PBS ductile polymer [22]. The tensile strength and the elongation at break of the MTPSCh/PBSE blends with 0.5–2.5% Er were higher compared to those of the MTPSCh/PBS blends. The increase in the tensile modulus, tensile strength, and elongation at break of the MTPSCh/PBSE samples was because of the interfacial crosslinking network between the CHG in MTPSCh and the epoxy groups in PBSE, the plasticization effect of Er, and the increase in interfacial compatibility between PBSE and MTPSCh. The formation of a crosslinking network because of the reaction between the epoxy group of Er with –NH of CHG and the plasticization effect of Er was reported previously [31,39]. However, the tensile properties of the TPSCh/PBSE5 blend decreased because of the excessive amount of Er which acted as a high plasticizer content.

### 3.3. Contact Angle

**Figure 3:**
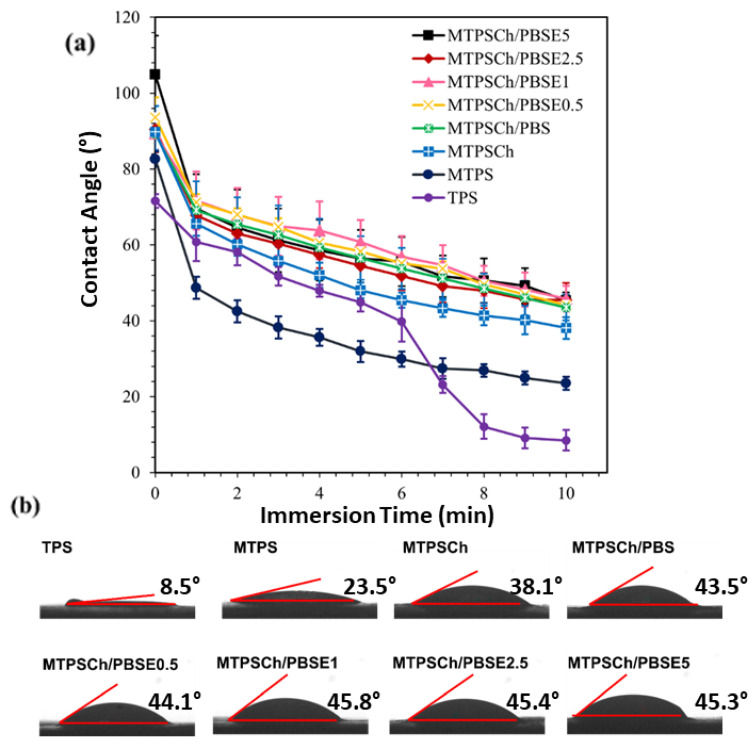
Water contact angle of TPS, MTPS, MTPSCh, MTPSCh/PBS, and MTPSCh/PBS with 0.5%, 1.0%, 2.5%, and 5.0% of Er; (**a**) water contact angle of samples and (**b**) image of water contact angle at 10 min.

The water contact angle is an indicator of surface tension, the hydrophilic and hydrophobic properties, and the water resistance of a material’s surface. The water contact angles of the TPS, MTPS, MTPSCh, MTPSCh/PBS, and MTPSCh/PBSE blends with 0.5%, 1.0%, 2.5%, and 5.0% Er are shown in Figure 3. A water droplet was absorbed onto the surface, and the contact angle was automatically recorded at 0, 2, 4, 6, 8, and 10 min after its absorption. TPS had the lowest contact angle after 10 min (8.5°), whereas MTPS showed at 23.5°. TPS exhibited higher hydrophilicity than MTPS. The addition of CHG to MTPS increased the contact angle at 10 min to 38.1° because of a reaction occurring in the crosslinking network of the MTPSCh blend. The crosslinking with TPS improved the water contact angle [34,38,45,46]. In the MTPSCh blend with PBS and PBSE at 0.5–5.0% Er, the water contact angle at 10 min increased to 43.5° (MTPSCh/PBS), 44.1° (MTPSCh/PBSE0.5), 45.8° (MTPSCh/PBSE1), 45.4° (MTPSCh/PBSE2.5), and 45.3° (MTPSCh/PBSE5), as shown in Figure 3b. The water contact angle was improved by the addition of PBS and PBSE to the MTPSCh blends. The addition of Er to PBS rarely improved the water resistance of the TPSCh/PBSE blends. The contact angles of polyethylene (67°), polypropylene (105°), polyethylene terepthalate (91°), and nylon (73°) have been reported [47,48]. The results indicated that the addition of PBS to TPSCh increased the interfacial tension of the MTPSCh/PBS blend, owing to the hydrophobicity of the PBS polymer. Er addition to PBSE slightly increases the contact angle and interfacial tension because of the occurrence of the crosslinking reaction via Er [43,49,50].

### 3.4. Scanning Electron Microscopy (SEM)

**Figure 4:**
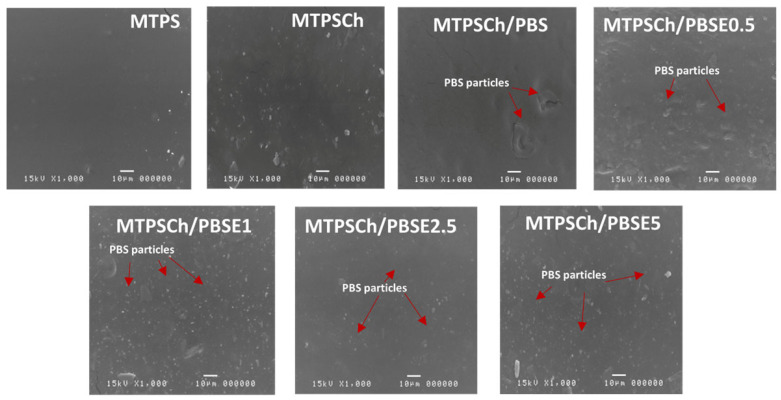
SEM images of MTPS, MTPSCh, MTPSCh/PBS, and MTPSCh/PBS with 0.5%, 1.0%, 2.5%, and 5.0% Er.

The microstructure and morphology of the polymer blend are closely related to the mechanical properties, processing ability, rheology, thermal properties, and water resistance. SEM was used to observe the microstructure of the MTPS and PBS blend. Figure 4 shows the SEM images of the fracture surfaces of MTPS, MTPSCh, MTPSCh/PBS, and MTPSCh/PBSE with 0.5–5.0% Er blends. MTPS showed a smooth fracture surface image, while MTPSCh exhibited an increase in the rough fracture surface because of the agglomeration of the crosslink phase between CHG and starch or glycerol [38]. The MTPSCh/PBS blend presented large PBS particles (10–15 μm) scattered in the MTPSCh, but the MTPSCh/PBSE with 0.5–2.5% Er had smaller white particle sizes (~1 μm). The small PBS particles indicated high interfacial adhesion between MTPSCh and PBSE owing to the addition of Er, the occurrence of the interfacial reaction, and a high shear rate of mixing [31,41]. The MTPSCh/PBSE2.5 exhibited small particles (~1 μm) with a smooth fracture surface, owing to the high interfacial adhesion between TPSCh and PBSE. However, excessive amounts of Er caused the particle size of MTPCh/PBSE5 to slightly increase. The high interfacial adhesion between MTPSCh and PBS via the reaction with Er resulted in a small particle size and smooth fracture surface morphology, which improved the tensile and water resistance properties of the blend. 

### 3.5. Thermal Stability

**Figure 5:**
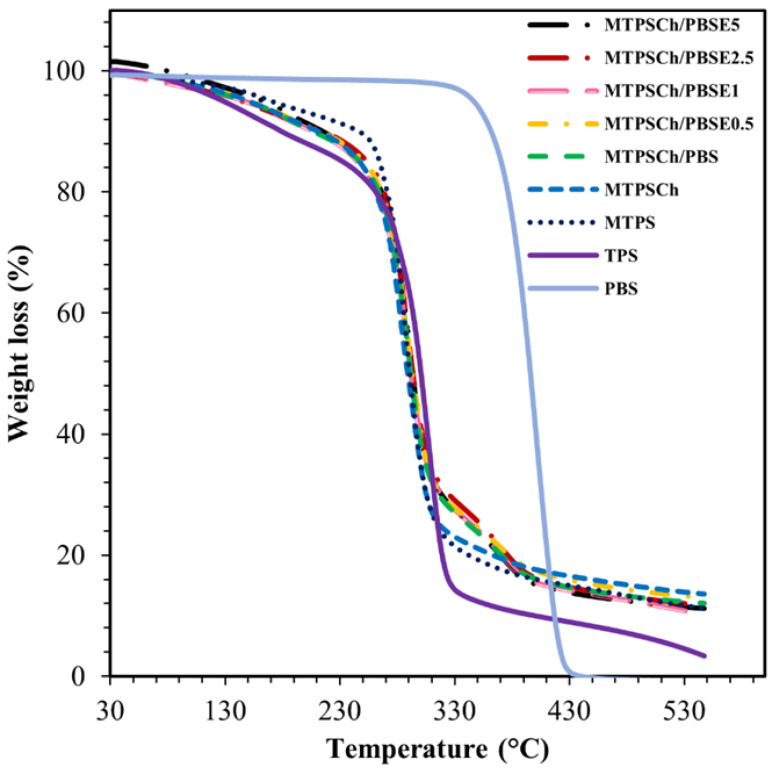
Thermograms of PBS, TPS, MTPS, MTPSCH1, MTPSCH1/PBS, and MTPSCH1/PBS with Er at 0.5%, 1.0%, 2.5%, and 5.0%.

The thermal gravimetric analysis was used to study phase decomposition, including various hydrates during heating. The thermal stabilities of PBS, TPS, MTPS, MTPSCh, MTPSCh/PBS, and MTPSCh/PBSE with 0.5–5.0% Er were studied using TGA, as shown in Figure 5. The thermal decomposition of PBS presented one stage in the thermogram at approximately 400 °C. The thermal decomposition of the TPS, MTPS, and MTPSCh blends showed two stages in the TGA thermograms. MTPS was a hydrophilic material which presented an –OH peak (3290 cm^−1^) in the FTIR spectra (Figure 1). The –OH groups of MTPS formed hydrogen bonding with moisture, holding water inside its structure. The first stage occurred during the evaporation of free water at approximately 100 °C by the water absorption of MTPS. The second stage was at 260–300 °C, which indicated the decomposition stage of starch [23]. The first and second decomposition stages of TPS showed the lowest weight loss owing to the low thermal stability of TPS. The MTPSCh, MTPSCh/PBS, and MTPSCh/PBSE blends showed less weight loss in the first stage compared to MTPS because of the decomposition of CHG of the blends. The MTPSCh samples with PBS and PBSE at 0.50%, 1.0%, 2.5%, and 5.0% showed thermal decomposition in three steps at 100–160 °C, 260–300 °C, and 320–380 °C owing to the evaporation of free water and CHG, the decomposition of starch, and the decomposition of Er and PBS in the samples, respectively [51]. The high weight loss% of the MTPSCh/PBS and MTPS/PBSE blends at 330 °C indicated a high thermal stability of PBS and the occurrence of crosslinking via the Er reaction. The MTPSCh/PBSE2.5 blend had the highest weight loss at approximately 330 °C because of the suitable crosslinking via the reaction with Er. TPS presented a low ash content at 330–530 °C due to the high degradation rate of the starch material at a high temperature, while PBS showed the lowest ash content from the degradation of ester groups in the PBS structure. MTPSCh showed more ash value than MTPS owing to the crosslinking of MTPS via the CHG reaction. The reaction between TPS and CHG was reported previously [35]. The MTPSCh/PBS and MTPSCh/PBSE samples exhibited almost the same ash content, which indicated the combination of the high degradation rate of PBS and the remaining crosslinking part inside MTPSCh.

## 4. Conclusions

Biodegradable starch-based blends were successfully developed through the melt blending of MTPSCh and PBS with Er. FTIR spectroscopy confirmed the reaction between the amino groups of CHG in MTPSCh and the epoxy groups of Er in PBSE. The tensile strength, elongation at break, morphology, water resistance, and thermal properties of the MTPSCh/PBSE blends were improved compared to TPS, especially at 2.5 wt% Er. Because of the high interfacial adhesion owing to the reaction and interaction of the blends, the MTPSCh/PBSE blends exhibited a morphology with small PBS particles distributed in an MTPS matrix. The addition of PBS and PBSE to the MTPSCh blends improved their thermal decomposition behavior. The reaction that occurred indicated an improvement of the compatibility of the MTPSCh/PBSE blend compared to MTPSCh/PBS, which improved the mechanical, water resistance, and thermal properties of the blends. Biodegradable polymers of MTPSCh/PBSE blends with good mechanical properties, thermal stability, and water resistance can be used in medical, agricultural, and packaging applications.

## Figures and Tables

**Table 1:** Code names and compositions of modified thermoplastic starch blend with chlorhexidine gluconate (MTPSCh), poly (butylene succinate) (PBS), and epoxy resin (Er) blends.

Samples		Composition (wt%/wt%)	
	MTPSCh	PBS	Er
PBS	-	100	-
MTPSCh	100	-	-
MTPSCh/PBS	90	10	-
MTPSCh/PBSE0.5	90	9.95	0.05
MTPSCh/PBSE1	90	9.90	0.10
MTPSCh/PBSE2.5	90	9.75	0.25
MTPSCh/PBSE5	90	9.50	0.50

## Data Availability

The data presented in this study are available upon request from the corresponding author.

## Abbreviations

CHG, chlorhexidine gluconate; Er, epoxy resin; FTIR, Fourier transform infrared spectroscopy; MTPS, modified thermoplastic starch; MTPSCh, modified thermoplastic starch blend with chlorhexidine gluconate; PBS, poly(butylene succinate); PBSE, the Er was melt-blended with PBS; SEM, scanning electron microscopy; TGA, thermogravimetric analysis.
